# SRTRL: Shared Radar-Communication Temporal Representation Learning for Beam Prediction in Vehicular and UAV mmWave Networks

**DOI:** 10.3390/s26206550

**Published:** 2026-10-15

**Authors:** Gang Wang, Qiwu Wu, Li Zhu, Tao Yang, Lingzhi Jiang

**Affiliations:** 1 School of Equipment Management and Support, Engineering University of PAP, Xi’an 710086, China; cli@hunnu.edu.cn (Q.W.); zhuli_cnrs@foxmail.com (L.Z.); yangtao_pap@163.com (T.Y.); 2 Key Laboratory of Counter-Terrorism Command & Information Engineering, Engineering University of PAP, Ministry of Education, Xi’an 710086, China; 3 School of Information Engineering, Engineering University of PAP, Xi’an 710086, China; jianglingzhi1123@ustb.edu.cn

**Keywords:** future beam prediction, GCN-TCN, multi-task learning, shared representation, millimeter-wave communication

## Abstract

Proactive beam management is essential for high-mobility millimeter-wave communication systems, where transmission latency may cause beams selected from current observations to become mismatched with the optimal beams at future transmission times. This paper investigates radar-only causal multi-horizon proactive beam prediction, aiming to directly rank future communication beams from past radar observations. To this end, we propose Shared-Beam-GCN-TCN, a multi-task framework that learns a shared predictive sensing-communication representation and performs structured decoding in the beam space. Specifically, four consecutive range-angle radar maps are encoded by a frame-wise convolutional neural network and a causal temporal convolutional network to capture short-term motion dynamics. The shared temporal representation is jointly optimized for future beam prediction and short-term radar prediction, encouraging the extraction of predictive sensing dynamics relevant to future communication states. A beam-space graph convolutional decoder then exploits the adjacency structure among 64 beam classes to model correlations between neighboring beams and improve future beam ranking. The framework is evaluated in two high-mobility mmWave communication scenarios, including a trajectory-disjoint DeepSense 6G vehicular scenario and a simulated unmanned aerial vehicle (UAV) scenario. In the vehicular scenario, Shared-Beam-GCN-TCN achieves the best overall performance across different prediction horizons, improving the average Top-3 and Top-5 accuracies by 8.2 and 6.9 percentage points over Seq-CNN-TCN, while reducing the mean beam-index error. In the UAV scenario, the proposed framework achieves an accuracy of 93.4% and a beam-index error of 1.022. These results demonstrate the effectiveness of shared predictive representation learning and structured beam-space decoding for causal multi-horizon beam prediction in high-mobility environments.

## 1. Introduction

### 1.1. Research Motivation and Background

Millimeter-wave (mmWave) communication systems rely on narrow directional beams to compensate for severe propagation loss and exploit the spatial gain of large antenna arrays [1,2]. In vehicular and aerial high-mobility scenarios, such as vehicle-to-infrastructure (V2I) and UAV-to-infrastructure (U2I) communications, the optimal beam may change rapidly because of mobility, blockage, line-of-sight variation, and dynamic propagation paths [3,4]. Although large antenna arrays provide substantial beamforming gain, they also enlarge the beam-search space and increase the sensitivity of the communication link to angular misalignment. Conventional exhaustive beam sweeping is robust but incurs considerable signaling and time-frequency overhead, particularly when the channel coherence time is comparable to the combined sensing, processing, signaling, and beam-switching delay [5,6].

Beam management is therefore not merely an instantaneous beam-classification, a cross-layer procedure involving beam measurement, reporting, refinement, tracking, and failure recovery [7,8]. During this sensing-to-actuation interval, a high-mobility user may move through a non-negligible angular range. Consequently, the beam identified from the latest observation may already be suboptimal when data transmission begins. This limitation motivates proactive beam prediction, where the objective is to infer the beam required at a future execution instant rather than recognize the beam associated with the current observation.

Radar-assisted beam management provides a promising means of reducing repeated communication-only beam training. Radar measurements contain range, angle, and motion-related information and can be acquired independently of the communication beam-training process. The evolution from shared radar-communication waveforms to integrated sensing and communication has further established the feasibility of exploiting sensing information for wireless resource management [9,10,11,12,13]. In particular, the DeepSense 6G platform provides synchronized real-world radar and mmWave measurements for evaluating sensing-assisted beam prediction [14]. Previous studies have shown that physically processed radar representations, such as range–angle and range–velocity maps, can support beam classification while reducing beam-training overhead [15].

### 1.2. Related Work and Research Gaps

Various forms of side information, including sub-6 GHz channels, position, camera images, LiDAR, and radar, have been used to reduce the mmWave beam-search space [16,17,18,19,20,21]. Out-of-band channel features can assist mmWave beam prediction, but they still consume radio resources and may be affected by frequency-dependent propagation mismatch [16,17]. Position- and vision-aided methods exploit user geometry and environmental context [18,19,20], while LiDAR-assisted approaches use sequential spatial observations to forecast future beam evolution [21]. Simulation platforms such as DeepMIMO have facilitated the development and benchmarking of learning-based mmWave beam-management algorithms [22]. Learning-based coordinated beamforming methods have further demonstrated the potential for reducing beam-management overhead in highly mobile systems [23].

Radar sensing is well suited to vehicular beam management, as it provides direct observations of surrounding geometry and motion and is largely unaffected by illumination conditions. Related studies have explored sensed geometry, radar odometry, pose information, communication-compatible radar waveforms, and environmental fingerprints for beam alignment and tracking [24,25,26,27]. Radar-assisted beam prediction has also been extended to joint beam selection and resource allocation and evaluated on larger real-world datasets [28,29]. However, most existing studies focus on single-step prediction or relatively short prediction horizons. The problem of predicting several future beams directly from a short radar sequence has received less attention, especially when training and test trajectories are strictly separated.

Temporal information in beam evolution has commonly been captured using recurrent neural networks or predictive beamforming architectures [30,31,32]. TCNs offer another way to model such dependencies through causal and dilated convolutions, with the advantages of parallel computation and an adjustable temporal receptive field [33,34]. Their effectiveness relative to recurrent architectures, however, has not been studied systematically for short radar observation sequences. This comparison is particularly relevant to multi-horizon prediction, where the encoder must extract useful motion patterns from only a limited history.

Another limitation is that conventional beam classifiers generally overlook the ordered structure of the beam codebook. Although GCNs can model relationships among neighboring beam candidates [35,36], their use for beam-space decoding remains limited. Moreover, since the ordered beam codebook can also be modeled using conventional local operators such as one-dimensional convolutions, it remains unclear whether the benefit of graph-based decoding arises from explicit beam-space topology or simply from increased decoder capacity. In addition, beam-label-only supervision may cause temporal encoders to rely on trajectory-specific or dataset-dependent correlations rather than genuine scene dynamics. Auxiliary supervision from future sensing observations can help preserve motion- and environment-related information in the learned representation.

Multi-task and predictive representation learning suggest that auxiliary future-state supervision can encourage a shared encoder to retain temporally persistent information [37,38,39]. However, improvements from an auxiliary task may result either from meaningful representation sharing or simply from increased network capacity. A rigorous evaluation should therefore compare shared and separate encoders under comparable task configurations, as well as graph-based and conventional local decoders with comparable model capacity. Furthermore, because practical beam management selects the number of candidate beams according to prediction confidence, the complete output distribution should be evaluated rather than relying solely on Top-1 accuracy [40].

### 1.3. Proposed Framework and Contributions

To address these limitations, this paper proposes Shared-Beam-GCN-TCN, a radar-assisted multi-horizon beam-prediction framework for proactive high-mobility mmWave communication systems, including terrestrial vehicular and aerial UAV scenarios. Consecutive range–angle radar maps are first processed by a frame-wise convolutional neural network and then encoded using a causal TCN. The resulting temporal representation is shared by a beam-prediction branch and an auxiliary future-radar-feature branch. A GCN defined over the beam codebook is further employed to model the relationships among neighboring beam classes. Based on the predicted probability distribution, an adaptive candidate-selection strategy adjusts the beam-search range according to model confidence. The CNN/TCN, graph convolution, and hard parameter-sharing mechanisms themselves are established techniques [33,34,35,36,37]; the contribution here is their task-specific coupling for radar-only causal multi-horizon beam prediction and the controlled evaluation used to isolate the roles of beam-space structure and shared predictive supervision. The main contributions are summarized as follows:

A radar-assisted multi-horizon framework for proactive beam prediction is developed. Unlike conventional methods that select beams from current observations, the proposed Shared-Beam-GCN-TCN directly predicts communication beams at multiple future horizons, thereby accounting for the sensing, inference, and beam-switching latency encountered in high-mobility millimeter-wave systems.

A shared temporal representation is learned through joint communication and radar-dynamics supervision. Consecutive range–angle radar maps are encoded using a frame-wise CNN and a causal TCN, and the resulting latent state is shared by the beam-prediction and future-radar-feature branches. The auxiliary radar task encourages the encoder to capture short-term environmental dynamics without relying on externally provided target positions or velocities.

A beam-space graph decoder and adaptive candidate-search strategy are introduced for efficient beam management. The graph convolutional decoder exploits the ordinal adjacency among the 64 beam classes to improve multi-horizon prediction, while adaptive candidate selection balances beam-search overhead against coverage.

## 2. System Model and Problem Formulation

We consider a high-mobility mmWave link between an infrastructure node equipped with radar and a phased-array transmitter and a mobile user (ground vehicle or UAV). The communication subsystem uses a fixed analog beam codebook, while synchronized communication measurements are used only offline to construct beam labels.

### 2.1. Radar-Communication System Model

Following the standard narrowband geometric beamforming formulation used in mmWave beam-management and predictive-beamforming studies [5,7,8,30], the channel at time t is written as:

(1) $$h_{t}=\sum_{l=1}^{L_{t}}\alpha_{\ell ,t}\;a\left(\vartheta_{\ell ,t},\varphi_{l,t}\right)$$

where, *L*_t_ is the number of effective propagation paths, α_*ℓ*,t_ ∈ ℂ is the complex gain of path *ℓ*, ϑ_l,t_ and φ_l,t_ are its azimuth and elevation angles relative to the BS array, and $a\left(\vartheta_{l,t},\varphi_{l,t}\right)\in C^{N_{a}}$ is the corresponding array response. Mobility, blockage, and multipath evolution make these quantities; consequently, the best beam can change even over short sampling intervals. The beam-index set and the analog beamforming codebook are defined as:

ℬ = {0, 1, …, *N_b_* − 1},  𝒞*_B_* = {**f***_n_*:*n* ∈ ℬ} (2)

Each $f_{n}\in C^{N_{a}}$ denotes one codebook beam and is normalized such that $∥f_{n}{∥}_{2}^{2}=1$. Accordingly, the beam quality associated with the beam **f***_n_* is represented as:

(3) $$\begin{array}{ccc} \gamma_{t,n}={\left|h_{t}^{H}f_{n}\right|}^{2}, & & n\in B \end{array}$$

and the supervised optimal-beam index is:

(4) $$y_{t}=\underset{n\in B}{\mathrm{argmax}}\;\;\gamma_{t,n}$$

This formulation is used only to define offline supervision; in the DeepSense experiment the same label can be obtained from synchronized beam-sweep receive powers. The deployed predictor does not require online CSI, a channel model, or exhaustive beam sweeping.

### 2.2. Radar-Derived Causal Representation Construction

The FMCW radar preprocessing follows the radar-aided beam-prediction pipeline in [15]. Rather than repeating the standard chirp, dechirping, radar-cube, and FFT derivations, we retain only the representation actually consumed by the learning model. After static-clutter suppression, range and angle Fourier transforms, and the chirp dimension is noncoherently averaged to obtain the range–angle map:

(5) $$R_{t}=\frac{1}{N_{c}}\sum_{a=0}^{N_{c}-1}\left|F_{\theta }\;\left(F_{r}\;\left(X_{t}^{c}\left(:,:,a\right)\right)\right)\right|$$

where, ℱ*_r_* and ℱ*_θ_* denote the range and angle transforms, respectively, and $R_{t}\in R_{\geq 0}^{N_{r}\times N_{\theta }}$, *N_c_* and *N*_θ_ denote the numbers of range and angle bins. This representation retains the spatial structure most directly related to directional beam selection while reducing the input dimensionality, consistent with real-world radar-aided beam prediction pipelines [15].

To control the large radar-amplitude dynamic range, each map is log-compressed and standardized using training-set statistics:

(6) $${\bar{R}}_{t}=\frac{\log\left(1+R_{t}\right)-\mu_{R}}{\sigma_{R}+\epsilon_{R}}$$

All operations are elementwise. The mean μ*_R_* and standard deviation σ*_R_* are estimated only from training trajectories to avoid leakage. A causal history of length *T* is constructed as (14):

(7) $$X_{t}=\left({\bar{R}}_{t-T+1},{\bar{R}}_{t-T+2},\ldots ,{\bar{R}}_{t}\right)$$

where, *ϵ_R_* > 0 is a small numerical-stability constant used in radar normalization. The experiments use *T* = 4. The history contains no observation after *t*, while displacement and intensity changes across frames provide implicit motion cues that are unavailable in a single snapshot.

### 2.3. Causal Multi-Horizon Beam Prediction Problem

The task is to infer several future beam distributions directly from the same causal radar history. Let H = {1,3,5} denote the prediction horizons. The trainable mapping is:

(8) $$\Psi_{\Theta }:X_{t}\mapsto {\left\{{\hat{p}}_{t+h}\right\}}_{h\in H}$$

where ℋ denotes the set of prediction horizons, *Θ* contains all trainable parameters and ${\hat{p}}_{t+h}\in {\left[0,1\right]}^{N_{b}}$ satisfies $\sum_{n=0}^{N_{b}-1}{\hat{p}}_{t+h,n}=1$. For each prediction horizon, a horizon-specific prediction head generates beam logits, which are normalized by a softmax function to obtain the beam probability distribution in (9). The corresponding Top-1 beam prediction is then obtained as (10).

(9) $${\hat{p}}_{t+h}=\mathrm{softmax}\left(g_{h}\left(X_{t};\Theta \right)\right)$$

(10) $${\hat{y}}_{t+h}=\underset{n\in B}{\mathrm{argmax}}\;{\hat{p}}_{t+h,n}$$

Retaining the full probability vector represents uncertainty among adjacent beams and enables Top-*K* candidate verification. A trajectory-disjoint training set with *M* samples is written as:

(11) $$D={\left\{\left(X_{t_{i}},{\left\{y_{t_{i}+h}\right\}}_{h\in H},{\bar{R}}_{t_{i}+1}\right)\right\}}_{i=1}^{M}$$

The next radar frame is included only as an auxiliary training target; it is never an online input. Trajectory-level partitioning is required because adjacent sliding windows share radar frames and random window-level splitting would cause temporal leakage.

## 3. Proposed Shared Radar-Dynamics Beam Graph Network

### 3.1. Architecture Overview and Proposed Algorithm

Figure 1 illustrates the overall architecture of the proposed Shared-Beam-GCN-TCN framework. The model integrates radar-based environmental perception, multi-horizon beam prediction, beam-space structural modeling, and auxiliary predictive representation learning within a unified multi-task architecture. Four consecutive range–angle radar maps are first processed by a weight-shared frame-level CNN to extract spatial features. These frame-wise embeddings are subsequently aggregated by a multi-scale causal TCN to capture short-term temporal evolution and generate a shared predictive state. In the communication branch, a beam-space graph convolution module exploits the ordinal adjacency among the 64 beam classes, followed by horizon-specific decoders that predict the optimal beams at future time steps t + 1, t + 3, and t + 5. In parallel, the radar perception branch predicts the feature representation of the next radar frame from the same latent state. During training, the target radar feature is generated by a frame encoder with shared CNN parameters and provides auxiliary supervision for the radar prediction task.The specific training and inference process is shown in Algorithm 1.
**Algorithm 1.** Shared Radar-Dynamics Beam Graph Training and Inference**Input:** causal radar history 𝒳*_t_*; future beam labels {*y_t_*_+*h*_}*_h_*_∈ℋ_$;\;\mathrm{training}-\mathrm{only}\;\mathrm{next}\;\mathrm{frame}\;{\bar{R}}_{t+1}$$;\;\mathrm{normalized}\;\mathrm{beam}\;\mathrm{graph}\;\hat{A}$. **1. Spatial encoding:**$\;\mathrm{apply}\;\mathrm{the}\;\mathrm{same}\;\mathrm{CNN}\;E_{\theta_{E}}$ to each frame in 𝒳*_t_* and stack the embeddings in temporal order. **2. Temporal encoding:**$\;\mathrm{apply}\;\mathrm{the}\;\mathrm{causal}\;\mathrm{dilated}\;\mathrm{TCN}\;T_{\theta_{T}}$ to obtain the shared dynamic state **z***_t_*. **3. Multi-horizon beam decoding:** for every *h* ∈ ℋ$,\;\mathrm{initialize}\;\mathrm{beam}-\mathrm{node}\;\mathrm{features}\;\mathrm{from}\;z_{t},\;\mathrm{propagate}\;\mathrm{them}\;\mathrm{over}\;\hat{A}$$,\;\mathrm{and}\;\mathrm{output}\;{\hat{p}}_{t+h}$. **4. Auxiliary prediction (training only):**$\;\mathrm{predict}\;\mathrm{the}\;\mathrm{next}-\mathrm{frame}\;\mathrm{radar}\;\mathrm{feature}\;\mathrm{from}\;z_{t}\;\mathrm{and}\;\mathrm{construct}\;\mathrm{the}\;\mathrm{target}\;\mathrm{from}\;{\bar{R}}_{t+1}$. **5. Joint update:** minimize the horizon-weighted beam loss, radar-feature prediction loss, and optional regularization; update the shared encoder and task-specific decoders. **6. Inference:** discard the auxiliary branch and output Top-1 or adaptive Top-*K* beam candidates from the horizon-specific probability distributions.

### 3.2. Spatiotemporal Radar Representation Learning

For the causal history 𝒳*_t_*, the shared encoder first processes each frame independently in space and then aggregates the resulting sequence in time:

(12) $$X_{t}\overset{E_{\theta_{E}}}{\to }\left(e_{t-T+1},\ldots ,e_{t}\right)\overset{T_{\theta_{T}}}{\to }z_{t}$$

where, $E_{\theta_{E}}$ is the shared frame encoder, $e_{\tau }\in R^{d_{e}}$ is a spatial embedding, $T_{\theta_{T}}$ is the causal temporal encoder, and $z_{t}\in R^{d_{z}}$ is the shared radar-dynamics state consumed by both the communication and auxiliary branches. The symbols *θ_E_* and *θ*_T_ denote all trainable parameters of the shared frame encoder and temporal encoder, respectively. The frame encoder uses conventional 2D convolutions and pooling, while the temporal encoder follows the causal dilated-convolution principle of TCNs [33,34]. We therefore omit the layer-by-layer convolution, zero-padding, and residual-block equations that are standard in those references. All four time positions share the same spatial parameters, and left-causal temporal padding ensures that the shared state **z***_t_* depends only on observations up to time t. The implemented channel, kernel, dilation, embedding, and pooling dimensions are reported in Table 1.

### 3.3. Codebook-Aware Multi-Horizon Beam Decoding

A conventional softmax head treats the *N_b_* beams as unrelated classes. In an ordered directional codebook, however, neighboring indices generally cover nearby or partially overlapping angular sectors. The proposed decoder represents each beam as a node and propagates evidence over a fixed beam-space graph. For the non-circular codebook used in this work, the adjacency matrix is

(13) $$A_{\mathrm{ij}}=\left\{\begin{array}{ll} 1, & \left|i-j\right|=1,\\ 0, & \mathrm{otherwise}, \end{array}\right.\;\;i,j\in B$$

This boundary rule does not connect the first and last beams. A circular distance should be used instead only when the physical codebook wraps around a full periodic field of view. Self-loops preserve each node’s own evidence, and symmetric normalization controls the feature scale:

(14) $$\begin{array}{ccccc} \tilde{A}=A+I_{N_{b}}, & & {\tilde{D}}_{\mathrm{ii}}=\sum_{j}{\tilde{A}}_{\mathrm{ij}}, & & \hat{A}={\tilde{D}}^{-1/2}\tilde{A}{\tilde{D}}^{-1/2} \end{array}$$

Different prediction horizons require different degrees of temporal extrapolation. Each horizon *h* ∈ ℋ therefore has its own node-initialization map, while all horizons use the same physical beam topology:

(15) $$q_{t,h}=W_{\mathrm{in},h}z_{t}+b_{\mathrm{in},h}\in R^{N_{b}d_{g}}$$

(16) $$H_{t,h}^{\left(0\right)}=\mathrm{reshape}\left(q_{t,h},N_{b},d_{g}\right)\in R^{N_{b}\times d_{g}}$$

where, the *n*th row of $H_{t,h}^{\left(0\right)}$ is the initial support assigned to node *n*. *d_g_* is the graph node-feature dimension (16 in Table 2), *L_G_* is the number of graph-convolutional layers (2), $\sigma_{g}^{\left(l\right)}$ is the graph activation, $1_{N_{b}}$ is the length-*N_b_* all-ones vector, and $W_{g,h}^{\left(l\right)}$ and $b_{g,h}^{\left(l\right)}$ are trainable graph-convolution parameters. Graph convolution then exchanges information only along the beam-node dimension:

(17) $$H_{t,h}^{\left(l+1\right)}=\sigma_{g}^{\left(l\right)}\;\left(\hat{A}H_{t,h}^{\left(l\right)}W_{g,h}^{\left(l\right)}+1_{N_{b}}{\left(b_{g,h}^{\left(l\right)}\right)}^{T}\right)$$

(18) $$o_{t,h}=H_{t,h}^{\left(L_{G}\right)}w_{o,h}+b_{o,h}1_{N_{b}}$$

The readout **o***_t_*_,*h*_ maps each beam-node feature to one scalar logit through trainable parameters **w***_o_*_,*h*_ and *b_o_*_,*h*_; softmax then converts these logits into the horizon-specific beam-probability vector. The TCN and GCN therefore model distinct relations: the TCN captures evolution along the radar time axis, whereas the GCN regularizes evidence along the ordered beam axis. This separation avoids conflating motion dynamics with label-space adjacency. A shallow graph decoder is used because the useful correlation is local; excessive graph depth would broaden the receptive neighborhood and can over-smooth directional evidence.

To adapt the candidate set to prediction uncertainty, the confidence score is defined as the maximum softmax probability, c = *max_b_*(*p_b_*), where *p_b_* denotes the predicted probability of beam *b*. The candidate number is selected from K ∈ {1, 3, 5} according to two confidence thresholds, *τ_high_* and *τ_low_*. Specifically, Top-1, Top-3, and Top-5 candidates are retained for high-, intermediate-, and low-confidence predictions, respectively. The thresholds are determined using the validation set and remain fixed during testing. In this way, the candidate set is adjusted according to prediction confidence rather than using a fixed search range for all samples.

### 3.4. Predictive Shared Representation Learning

The complete forward path is CNN → TCN → GCN. Let $\Phi_{\theta_{E},\theta_{T}}$ denote the shared spatial-temporal encoder and let $B_{h,\theta_{B,h}}$ denote the horizon-specific graph decoder. Their end-to-end coupling is

(19) $${\hat{p}}_{t+h}=B_{h,\theta_{B,h}}\;\left(\Phi_{\theta_{E},\theta_{T}}\left(X_{t}\right)\right)$$

(20) $$B_{h,\theta_{B,h}}=\mathrm{Softmax}\circ O_{h}\circ G_{h,\hat{A}}\circ P_{h},\;\;h\in H$$

For a mini-batch of B samples, the horizon-weighted beam-classification loss is:

(21) $$\begin{array}{ccc} L_{\mathrm{beam}}^{\left(h\right)}=-\frac{1}{B}\sum_{i=1}^{B}\log\;\left({\hat{p}}_{i,h,y_{i,h}}\right), & & h\in H \end{array}$$

(22) $$L_{\mathrm{beam}}=\sum_{h\in H}\alpha_{h}L_{\mathrm{beam}}^{\left(h\right)}\;$$

(23) $$\begin{array}{ccc} \alpha_{h}\geq 0, & & \sum_{h\in H}\alpha_{h}=1 \end{array}$$

The radar-dynamics objective is formulated as the mean squared feature-prediction error. The target next-frame radar feature is directly extracted from the measured radar observation at the future timestamp and is treated as a fixed supervision signal during training. Therefore, no gradient is propagated through the target feature generation process. Before calculating the loss, the radar features are normalized to reduce the influence of feature-scale variation, for sample i, ${\hat{f}}_{R}^{i}$, and $f_{R}^{i}$ denote the predicted and target next-frame radar features.

(24) $$L_{\mathrm{rad}}=\frac{1}{Bd_{e}}\sum_{i=1}^{B}{∥{\hat{f}}_{R}^{i}-f_{R}^{i}∥}_{2}^{2}$$

The full training objective combines the two tasks and optional weight regularization:

ℒ = ℒ_beam_ + *λ*_rad_ℒ_rad_  (25)

The distinction between the proposed shared model and the separate-encoder control is expressed directly by the gradient acting on the common encoder parameters *θ_S_* = {*θ_E_*,*θ_T_*}. In the separate-encoder variant, the temporal encoders for beam prediction and future-radar prediction are independently optimized, and no temporal encoding parameters are shared between the two tasks. In contrast, the proposed framework employs a shared temporal representation, allowing the two objectives to jointly optimize the encoder parameters.

(26) $$\nabla_{\theta_{S}}L=\sum_{h\in H}\alpha_{h}\nabla_{\theta_{S}}L_{\mathrm{beam}}^{\left(h\right)}+\lambda_{\mathrm{rad}}\nabla_{\theta_{S}}L_{\mathrm{rad}}$$

The beam terms promote communication-discriminative features, whereas the radar term promotes sensitivity to short-term scene evolution. In the separate-encoder control, these gradients act on disjoint temporal parameters, preventing the radar task from shaping the representation used by the beam decoder. The coefficient *λ*_rad_ is selected on validation trajectories to avoid negative transfer. The coefficients *λ*_rad_ and *λ*_reg_ control the auxiliary radar loss and L2 regularization, respectively.

## 4. Materials and Methods

### 4.1. Dataset Configuration and Sequence Construction

The proposed framework targets high-mobility mmWave communication scenarios, including terrestrial vehicles and aerial platforms. In this work, Scenario 9 of the DeepSense 6G dataset [14,15] is adopted as the primary evaluation benchmark, which provides synchronized radar measurements and mmWave beam labels for vehicle-to-infrastructure (V2I) communication. This scenario contains realistic mobility, blockage effects, and channel variations. The same radar-assisted prediction framework can also be applied to UAV communication scenarios, where mobility-induced angular variations introduce similar beam alignment challenges. Each radar observation is converted into a range-angle representation, and the corresponding beam label denotes the optimal beam at the same acquisition instant.

As shown in Figure 2, experiments are conducted under two representative high-mobility mmWave scenarios: the real-world DeepSense 6G Scenario 9 vehicular dataset and the controlled synthetic UAV dataset. Samples are kept in their original temporal, and complete trajectories are assigned to training, validation, or test sets before any sliding windows are generated. A one-frame sliding-window stride is used in both scenarios. Four consecutive range-angle maps form each input, and future beams are predicted at h = 1, 3, and 5. Table 2 summarizes the corresponding physical timing, trajectory allocation, and sample counts.

For DeepSense 6G Scenario 9, the sequence index supplied with the dataset is used as the vehicle-run identifier. The official Scenario 9 metadata report 5964 synchronized multimodal instances and an average data-capture rate of 7.67 frames/s [14], corresponding to a mean synchronized radar/communication interval of approximately 0.130 s. Complete sequence-indexed runs are assigned to training, validation, or test subsets before temporal-window generation, yielding 3486/670/720 windows, respectively. Because four input frames span three inter-frame intervals, the history covers approximately 0.391 s from the earliest to the latest observation. The h = 1, 3, and 5 labels therefore correspond to mean lead times of approximately 0.130, 0.391, and 0.652 s. The original timestamps are not uniform, so these values should be interpreted as average physical intervals rather than exact delays for every sample. Complete sequence-indexed vehicle runs are partitioned prior to sliding-window sample generation. For the vehicular DeepSense 6G Scenario 9 dataset, the trajectory-disjoint partition assigns 22 full runs to training, 5 full runs to validation, and 6 full runs to testing. No vehicle run is shared between subsets, preventing temporal leakage even when sliding-window samples are subsequently generated. The resulting temporal sliding-window samples are 3486 (train), 670 (validation), and 720 (test).

To ensure reproducibility and a fair comparison, all temporal learning-based models were trained under a consistent optimization protocol using the Adam optimizer for 80 epochs, with an initial learning rate of 5 × 10^−4^, a weight decay of 1 × 10^−4^, and a batch size of 32. The learning rate was halved after 20 epochs, the random seed was fixed to 0, and the checkpoint yielding the lowest validation loss was retained for testing. The main hyperparameters were determined through preliminary experiments on the validation set and were fixed before test-set evaluation. Cross-entropy loss was adopted for beam classification, while the auxiliary future-radar prediction task in Shared-Beam-GCN-TCN used with a weight of 0.2. For the synthetic UAV evaluation, the dataset contains 72 trajectory-disjoint sequences, each consisting of 80 radar frames sampled at 10 Hz. These trajectories were divided into 50, 11, and 11 sequences for training, validation, and testing, respectively, resulting in 3600, 792, and 792 temporal samples. Each sample takes four consecutive radar frames as input to predict the optimal beam at horizons h = 1, 3, and 5, which correspond to lead times of 0.1, 0.3, and 0.5 s given the 0.1-s frame interval.

### 4.2. Synthetic UAV Scenario and Simulation Configuration

To evaluate the proposed framework under aerial mobility, a controlled synthetic UAV-to-infrastructure scenario was constructed. The dataset contains 72 trajectories covering straight flight, orbiting, and vertical climb/descent, with 24 trajectories for each motion pattern. Each trajectory contains 80 radar frames sampled at 10 Hz, yielding 5760 frames in total. The base-station height is 10 m, while the UAV altitude and speed range from approximately 46.9 to 114.2 m and 2.18 to 24.11 m/s, respectively. A fixed random seed of 7 is used for reproducibility.

Each radar observation is represented by a 256 × 64 range–angle map covering 10–250 m in range and −60° to 60° in azimuth. The synthetic maps include the UAV return, reflected components, static scatterers, range-dependent attenuation, and Rayleigh-distributed background noise. The beam label is obtained by uniformly quantizing the relative UAV azimuth into 64 sectors over the same angular range. Adjacent beam indices therefore correspond to neighboring azimuth sectors, and the beam graph connects nearby indices within a neighborhood width of two. Although the trajectories include three-dimensional motion and altitude variation, the beam labels depend only on azimuth. Altitude changes affect the radar observations but do not directly determine the beam index. Elevation-dependent beam steering, a specific antenna-array geometry, and a full mmWave channel are not explicitly modeled. The UAV experiment is therefore intended as a controlled evaluation of temporal beam prediction under aerial mobility rather than a full three-dimensional communication-system simulation.

Four consecutive radar observations are used to predict the beam index h = 1, 3, and 5 frames ahead, corresponding to 0.1, 0.3, and 0.5 s, respectively. To avoid trajectory leakage, complete trajectories are separated across training, validation, and test sets, with 50 trajectories (3600 temporal samples), 11 trajectories (792 samples), and 11 trajectories (792 samples), respectively. Seq-CNN-TCN and Beam-GCN-TCN are trained using Adam with a batch size of 16 and an initial learning rate of 10^−3^. Shared-Beam-GCN-TCN is initialized from the trained UAV Beam-GCN-TCN model and fine-tuned with a batch size of 32 and a learning rate of 5 × 10^−4^, with the auxiliary future-radar loss weighted by 0.2. It is worth clarifying the fairness of comparison for UAV scenario baselines. Seq-CNN-TCN and Beam-GCN-TCN are trained from random initialization with batch size 16 and initial learning rate of 10^−3^. For Shared-Beam-GCN-TCN, the pre-trained Beam-GCN-TCN provides initialization for the shared frame-CNN, TCN, and beam-GCN decoder parameters, while the newly added radar-auxiliary prediction head is randomly initialized. All UAV models keep identical backbone dimensions, beam-graph configuration, input representation, prediction horizons, trajectory-disjoint data split, and validation-loss-driven checkpoint selection rule. All models are tested on exactly the same held-out test flight trajectories. Validation-set ablation confirms that training Shared-Beam-GCN-TCN from scratch produces consistent performance trends; fine-tuning mainly stabilizes multi-task convergence. Performance improvements are attributed to the auxiliary radar-prediction multi-task objective and shared temporal representation learning, rather than weight initialization. Validation performance is used for model selection before final evaluation on the held-out test trajectories.

### 4.3. Radar Representations and Baselines

For completeness, the reported Top-K accuracy and mean beam-index error are computed over all test samples and prediction horizons as:

(27) $${\mathrm{Acc}}_{K}=\frac{1}{N\left|H\right|}\sum_{i=1}^{N}\sum_{h\in H}I\;\left[y_{i,h}\in \mathrm{TopK}\left(p^{i,h},K\right)\right]$$

(28) $$E_{\mathrm{beam}}=\frac{1}{N\left|H\right|}\sum_{i=1}^{N}\sum_{h\in H}\left|{\hat{y}}_{i,h}-y_{i,h}\right|$$

where *N* is the number of test samples, *y_i_*_,*h*_ is the ground-truth beam index, and ${\hat{y}}_{i,h}$ is the Top-1 predicted beam index. These definitions explicitly evaluate both candidate coverage and ordinal beam-index locality.

Range-angle is the primary representation. Radar Cube (RC) and range-velocity (RV) inputs are retained for single-frame comparison. Figure 3 illustrates the radar preprocessing chain and a representative range-angle beam-probability output.

To evaluate the proposed Shared-Beam-GCN-TCN from both performance and architectural perspectives, we compare it with representative heuristic and learning-based baselines, together with controlled variants designed to isolate the effects of beam-space graph modeling, auxiliary radar prediction, and shared representation learning. The evaluated methods are as follows:

Beam Persistence: a non-learning heuristic baseline that predicts the current beam and its adjacent beams as future candidates, following the temporal-continuity assumption commonly adopted in predictive beamforming and beam-tracking methods [5].

RC-CNN, RV-CNN, and RA-CNN: single-frame convolutional neural network classifiers using the radar cube, range–velocity map, and range–angle map as inputs, respectively. These radar representations and their application to radar-assisted beam prediction follow the processing framework introduced in [15].

Seq-CNN-GRU and Seq-CNN-TCN: sequential models that encode four consecutive range–angle maps using a shared CNN frame encoder, followed by either a gated recurrent unit (GRU) or a causal temporal convolutional network (TCN) [31].

Beam-GCN-TCN: an extension of Seq-CNN-TCN in which the temporal representation is decoded through a beam-space graph convolutional network. The graph decoder is based on the graph convolutional formulation proposed in [35] and exploits adjacency among ordered beam classes.

TCN + RadarAux: a temporal model trained jointly for future-beam classification and next-frame radar-feature prediction, but without the beam-space graph. The auxiliary learning strategy follows the general principle of shared-representation multi-task learning [37].

Beam-GCN-TCN + SepEnc: a control model incorporating both the beam-space graph decoder and the radar auxiliary task while assigning separate temporal encoders to the two branches. This configuration is used to distinguish the effect of representation sharing from the effect of simply adding an auxiliary task.

Shared-Beam-CNN-TCN: a controlled variant of the proposed model in which the beam-space GCN decoder is replaced by a parameter-comparable 1D convolutional decoder. All other components and training settings remain unchanged, enabling a direct assessment of graph-based beam-space modeling against conventional local convolution.

Shared-Beam-GCN-TCN: the proposed full shared-encoder multi-task model.

## 5. Results

### 5.1. Multi-Horizon Future Beam Prediction

Table 3 compares the benchmark methods across the three prediction horizons. Beam Persistence performs reasonably at the shortest horizon but deteriorates rapidly as the prediction horizon increases, indicating that relying solely on the current beam state provides limited predictive capability in high-mobility scenarios. In comparison, the learning-based methods exhibit substantially more stable performance across different horizons. Among the benchmark models, Seq-CNN-TCN achieves the highest Top-1 accuracy at all three prediction horizons, reaching 0.369, 0.399, and 0.378 for h = 1, 3, and 5, respectively. This indicates that temporal feature extraction is particularly beneficial for identifying the most likely future beam. The CNN-based baselines also provide competitive performance, with RA-CNN generally outperforming the other conventional CNN variants.

In contrast, Beam-GCN-TCN consistently achieves the best Top-3 and Top-5 performance among the benchmark methods. Its Top-5 accuracies reach 0.867, 0.860, and 0.832 at h = 1, 3, and 5. This suggests that beam-space graph propagation is more effective in preserving structural correlations among neighboring beam candidates, thereby improving candidate-beam coverage even when the exact Top-1 prediction is not selected.

### 5.2. Ablation Study of Shared Temporal Representation

To clarify the individual and joint contributions of radar auxiliary supervision, beam-space graph decoding, and shared temporal encoding, Table 4 compares several controlled variants. TCN + Radar Aux. retains the auxiliary future-radar prediction task without the graph decoder, whereas Beam-GCN-TCN uses the graph decoder without radar auxiliary supervision. Beam-GCN-TCN + Sep. Enc. incorporates both components but assigns independent temporal encoders to the two tasks. Shared-Beam-CNN-TCN further replaces the graph decoder with a parameter-comparable 1D convolutional decoder while retaining the shared encoder and auxiliary task, providing a controlled comparison between conventional local convolution and graph-based beam-space modeling. The proposed Shared-Beam-GCN-TCN combines all three components within a shared representation.

Beam-GCN-TCN consistently outperforms TCN + Radar Aux., showing that auxiliary radar prediction alone cannot replace explicit modeling of neighboring beam relationships. Adding the auxiliary task with separate encoders further improves most metrics, indicating the value of motion-related supervision. Compared with the parameter-comparable Shared-Beam-CNN-TCN, Shared-Beam-GCN-TCN improves the average Top-1, Top-3, and Top-5 accuracies by approximately 1.3, 0.9, and 0.7 percentage points, respectively, suggesting that the gain also benefits from explicit beam-space adjacency modeling.

Comparing Shared-Beam-GCN-TCN with Beam-GCN-TCN + Sep. Enc. further isolates the effect of temporal representation sharing. Averaged across the three horizons, shared encoding improves Top-1, Top-3, and Top-5 accuracy by approximately 0.8, 1.4, and 1.1 percentage points, respectively.

To further assess the robustness of the proposed framework, four representative models were evaluated across five random seeds under identical experimental settings. As summarized in Table 5 and illustrated in Figure 4a,b, Shared-Beam-GCN-TCN consistently achieves the highest Top-5 accuracy and adaptive coverage, with limited variations across repeated runs. Compared with Seq-CNN-TCN and Beam-GCN-TCN, the proposed model maintains clear performance advantages, while shared temporal encoding provides additional improvements over separate encoding. Figure 4c further presents the paired performance differences and their 95% confidence intervals, indicating consistent gains over the competing models. Although the improvements over the separate-encoder variant are relatively modest, these results provide further evidence of the effectiveness and stability of shared temporal representation learning.

### 5.3. Overall Performance and Confidence-Adaptive Beam Search

Table 6 summarizes the average performance of the compared methods over the three prediction horizons. In addition to conventional Top-K accuracy and beam-index error, confidence-adaptive candidate-selection performance is reported to evaluate the trade-off between candidate-beam retention and candidate-set size. Beam-index error reflects the deviation between the predicted and ground-truth beam indices, while adaptive accuracy measures whether the ground-truth beam is retained within the confidence-dependent candidate set. Together with the average number of searched beams, these metrics provide a prediction-level assessment of how effectively the model concentrates the search on a compact set of likely beam candidates.

Shared-Beam-GCN-TCN achieves the best overall prediction performance, with average Top-1, Top-3, and Top-5 accuracies of 0.395, 0.735, and 0.892, respectively, and the lowest mean beam-index error of 1.271. Compared with Seq-CNN-TCN, it improves Top-3 and Top-5 accuracy by 8.2 and 6.9 percentage points, respectively, while reducing the beam-index error by 11.3%. Although the improvement in Top-1 accuracy is relatively modest, the more pronounced Top-k gains indicate better candidate-beam coverage and beam localization. These improvements suggest that shared radar-temporal representations, beam-space structural learning, and auxiliary future-radar prediction provide complementary information for identifying and ranking candidate beams.

The advantage is further reflected in the adaptive beam-search results. Shared-Beam-GCN-TCN achieves an adaptive candidate accuracy of 0.844 with an average of 4.019 beam evaluations, corresponding to approximately 6.3% of the 64-beam codebook. Compared with Beam-GCN-TCN + Sep. Enc., shared encoding slightly improves adaptive accuracy from 0.839 to 0.844 while reducing the average search size from 4.167 to 4.019 beams. Since both models incorporate the same auxiliary task and beam-space graph decoder, these results suggest that sharing temporal representations between the two prediction tasks provides a modest but consistent benefit for beam prediction and adaptive candidate selection.

Since both models already include the radar auxiliary task and the beam-space graph decoder, this difference can mainly be attributed to the shared temporal representation. With shared features, the confidence scores become more informative for candidate selection, so the search policy can retain fewer beams without reducing coverage. The gain from feature sharing thus carries over from conventional prediction metrics to the practical beam-search stage.

Figure 5 compares the Top-kperformance of all methods across the three prediction horizons. The proposed Shared-Beam-GCN-TCN achieves the strongest overall performance, particularly in Top-3 and Top-5 accuracy, indicating more reliable retention of the correct beam within a compact candidate set. As the prediction horizon increases, its candidate-beam coverage remains relatively stable, with Top-5 accuracy decreasing only from 90.3% at h = 1 to 87.6% at h = 5. In contrast, Beam Persistence deteriorates sharply at longer horizons, while the other benchmark methods generally exhibit lower candidate coverage and greater horizon-dependent variation.

Figure 6 compares the row-normalized confusion matrices of Shared-Beam-GCN-TCN, Beam-GCN-TCN, and Beam Persistence at h = 5. Shared-Beam-GCN-TCN exhibits a strong concentration around the main diagonal, indicating that most predictions remain close to the ground-truth beam indices even at the longer prediction horizon. Its Top-5 accuracy reaches 87.64%, compared with 83.19% for Beam-GCN-TCN and 4.03% for Beam Persistence. Beam-GCN-TCN also maintains a clear diagonal structure but exhibits more off-diagonal deviations, while Beam Persistence shows a noticeably shifted prediction distribution, reflecting its limited ability to capture future beam changes. These results suggest that combining shared radar-temporal representations with beam-space structural modeling improves candidate-beam localization and reduces prediction deviations under longer prediction horizons.

### 5.4. Radar-Dynamics and Representation Analysis

Table 7 compares the future radar-feature prediction and beam-retrieval results. The shared radar predictor achieves an average cosine similarity of 0.959 with the ground-truth future features, higher than linear feature extrapolation (0.917) but slightly below current-feature persistence (0.966). The high similarity of the persistence baseline is reasonable, since consecutive radar observations retain substantial static background and echo structure. Therefore, feature-level similarity alone does not fully reflect whether the predicted representation contains information useful for future beam selection.

A clearer difference appears in the beam-retrieval results. The shared radar predictor reduces the mean beam-index error to 1.73, compared with 1.90 for current-feature persistence and 2.17 for linear extrapolation, while achieving the highest within-one-beam accuracy of 55.08%. Although current-feature persistence preserves the overall radar representation well, it is less effective at capturing directional changes related to future beam evolution. The shared predictor better retains these temporal variations, leading to retrieved beam indices that are closer to the ground-truth future beams.

Figure 7 provides a qualitative visualization of the learned temporal representations using UMAP [40]. When colored by the future beam index, both Beam-GCN-TCN and Shared-Beam-GCN-TCN exhibit a smooth transition among neighboring beam classes, indicating that the latent space preserves the ordinal continuity of the beam codebook. When colored by radar dynamic change, the shared model shows a clearer organization of samples associated with different levels of temporal variation, suggesting that the auxiliary radar-prediction task encourages the latent representation to retain motion-related information. Overall, these observations indicate that the shared representation jointly captures beam-space structure and short-term radar dynamics. Nevertheless, the UMAP results are intended only as qualitative evidence and should not be interpreted as establishing a causal relationship.

### 5.5. Applicability Evaluation in a Synthetic UAV Scenario

Based on the synthetic UAV configuration described in Section 4.2, the proposed framework is further evaluated under three representative aerial mobility patterns. UAV trajectories involve both horizontal displacement and altitude variation, introducing additional changes in propagation geometry and beam direction compared with terrestrial vehicle motion. The experiment therefore provides a complementary evaluation of radar-assisted temporal beam prediction under aerial mobility. Since the UAV models are independently trained and evaluated on the synthetic UAV dataset, the results demonstrate scenario applicability rather than cross-domain generalization.

The UAV scenario is generated around an infrastructure node equipped with a radar sensing module and a communication beam-selection module. The UAV follows three representative flight patterns, including straight-line flight, horizontal turning/orbiting, and vertical maneuvering, to capture diverse aerial mobility behaviors. During each trajectory, radar observations and communication beam labels are generated synchronously. The received radar signals are converted into range–angle maps using the same preprocessing procedure as in the vehicular experiment. For beam-label generation, the relative azimuth angle between the UAV and the infrastructure node is uniformly quantized over the predefined field of view into 64 azimuth sectors, and the corresponding sector index is used as the beam label. Thus, the UAV experiment adopts an azimuth-based beam-labeling scheme rather than an explicit antenna-array or channel-level beamforming simulation. Although the trajectories include altitude variations, elevation-dependent beam steering is not considered in the current setup.

The constructed UAV dataset contains 72 flight trajectories with synchronized radar-beam sequences. Each trajectory is divided into sliding-window samples using four consecutive radar frames as input, and the future beam prediction tasks are defined at horizons of h = 1, 3, and 5. To avoid temporal leakage, the dataset is partitioned according to complete flight trajectories, ensuring that trajectories appearing in the training set do not overlap with those in validation or test sets. The detailed configurations of the vehicular and UAV scenarios are summarized in Table 1.

Figure 8 illustrates representative UAV flight trajectories and the corresponding beam-index variations under different mobility patterns. Three typical flight modes are considered, including straight flight, horizontal orbiting, and vertical maneuvering, which introduce different spatial evolution characteristics. As shown in Figure 8b, the optimal beam index varies with the UAV trajectory, and different mobility patterns result in distinct beam transition behaviors. In particular, vertical movement changes the elevation geometry between the UAV and the infrastructure node, leading to beam variations that cannot be fully captured by horizontal displacement alone. These results indicate the importance of temporal sensing information for predicting future beams in aerial communication scenarios.

For the UAV evaluation, two representative baselines, Seq-CNN-TCN and Beam-GCN-TCN, are selected to compare the effects of temporal modeling and beam-space structural modeling. Since this experiment aims to evaluate the scenario applicability under aerial mobility of the proposed representation learning framework rather than establish a separate UAV benchmark, only the key temporal and graph-based baselines are considered. The same network architecture, training objective, and prediction configurations are adopted without any UAV-specific modifications.

Table 8 summarizes the average future-beam prediction performance in the UAV scenario. Compared with Seq-CNN-TCN, Beam-GCN-TCN improves the Top-1 and Top-5 accuracies from 0.2652 and 0.8822 to 0.2782 and 0.9242, respectively, demonstrating the benefit of exploiting local correlations among neighboring beam candidates. With the shared radar-temporal representation learning, Shared-Beam-GCN-TCN further improves the prediction performance and achieves Top-1, Top-3, and Top-5 accuracies of 0.3123, 0.7908, and 0.9339, respectively. Compared with Seq-CNN-TCN, the proposed method increases Top-1 accuracy by 4.71 percentage points and reduces the beam-index error by 24.1%. The consistent improvements under UAV mobility indicate that the learned radar-temporal representation can also capture beam evolution patterns in three-dimensional aerial communication scenarios.

## 6. Discussion

The experimental results show that multi-horizon beam prediction benefits from jointly exploiting temporal radar information and beam-space structural correlations. Sequential models generally provide stronger future-beam prediction than methods relying primarily on instantaneous observations, highlighting the importance of short-term mobility information. By explicitly modeling adjacency relationships among neighboring beam classes, Shared-Beam-GCN-TCN further improves candidate-beam ranking and overall prediction performance. Compared with Seq-CNN-TCN, the proposed framework improves the average Top-3 and Top-5 accuracies by 8.2 and 6.9 percentage points, respectively, while reducing the mean beam-index error by 11.3%. The ablation results further show that the shared-encoder architecture consistently outperforms its separate-encoder counterpart, indicating that future-radar supervision is more effective when directly coupled with the beam-prediction task through a shared temporal representation.

The radar representation analysis and adaptive beam-search evaluation provide further insight into the learned features. Although current-feature persistence yields slightly higher feature-level cosine similarity, the shared radar-prediction branch achieves lower beam-index retrieval error and better local beam consistency. This suggests that the learned representation preserves temporal variations associated with future beam evolution rather than simply retaining static radar structures. In addition, the adaptive search strategy achieves 84.4% candidate-beam coverage with only 4.019 searched beams on average, indicating that the predicted beam distribution can substantially reduce the candidate set while retaining a high proportion of the target beams. Nevertheless, the current evaluation is based on a fixed beam codebook and a limited set of mobility scenarios, and further validation is needed across different environments, hardware platforms, and real-time deployment conditions.

Beyond terrestrial vehicular communication, the UAV experiment further evaluates the applicability of the proposed framework under aerial mobility. Using the same model architecture, Shared-Beam-GCN-TCN maintains favorable performance relative to the temporal and graph-based baselines, achieving a Top-1 accuracy of 0.3123 and a beam-index error of 1.022 in the simulated UAV scenario. These results suggest that the shared radar-temporal representation can capture mobility-related beam evolution under both terrestrial and aerial trajectory patterns. The UAV evaluation therefore provides additional evidence of the applicability of radar-assisted beam prediction to broader high-mobility mmWave scenarios, while more realistic channel, antenna-array, and elevation-aware modeling remains an important direction for future study.

It is worth noting that the individual components do not improve all evaluation metrics monotonically. The auxiliary radar-prediction task slightly degrades the basic TCN when introduced alone, suggesting possible competition between preserving temporal sensing dynamics and learning discriminative features for exact beam identification. Similarly, the beam-space GCN improves Top-3 and Top-5 accuracy by modeling correlations among neighboring slightly reduces Top-1 accuracy and increases the mean beam-index error, possibly due to feature smoothing between adjacent classes. The complete Shared-Beam-GCN-TCN mitigates these trade-offs by jointly learning temporal dynamics and beam-space correlations, allowing the auxiliary task and graph structure to provide complementary information for future beam prediction.

## 7. Conclusions

This paper proposed Shared-Beam-GCN-TCN, a radar-assisted multi-task framework for proactive multi-horizon beam prediction in high-mobility millimeter-wave communication systems. The framework integrates temporal radar representation learning, beam-space graph decoding, and auxiliary future-radar supervision to exploit short-term mobility information and structural correlations among neighboring beam candidates. Experiments on the trajectory-disjoint DeepSense 6G dataset demonstrate robust performance across prediction horizons of h = 1, 3, 5. Averaged over the three horizons, Shared-Beam-GCN-TCN improves Top-3 and Top-5 accuracies by 8.2 and 6.9 percentage points, respectively, over Seq-CNN-TCN, while reducing the mean beam-index error by 11.3%. The confidence-adaptive candidate selection further achieves 84.4% candidate-beam coverage with only 4.019 searched beams on average, demonstrating its ability to reduce the candidate set while retaining a high proportion of the target beams. Additional evaluation in a simulated UAV scenario further demonstrates the applicability of the same framework under aerial mobility without UAV-specific architectural modifications. Future work will extend the framework to more diverse mobility environments and incorporate realistic channel, antenna-array, and real-time deployment considerations.

## Figures and Tables

**Figure 1:**
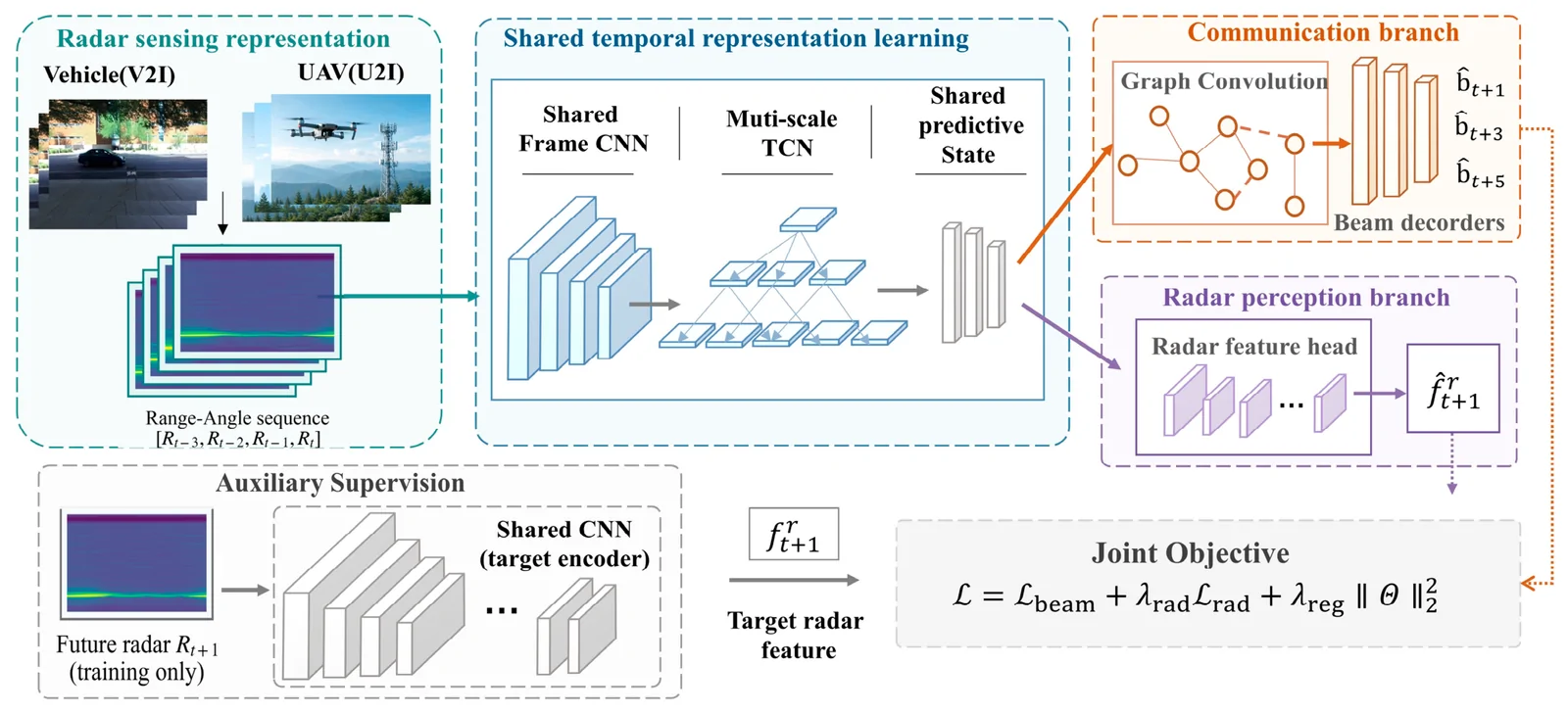
Architecture of Shared-Beam-GCN-TCN.

**Figure 2:**
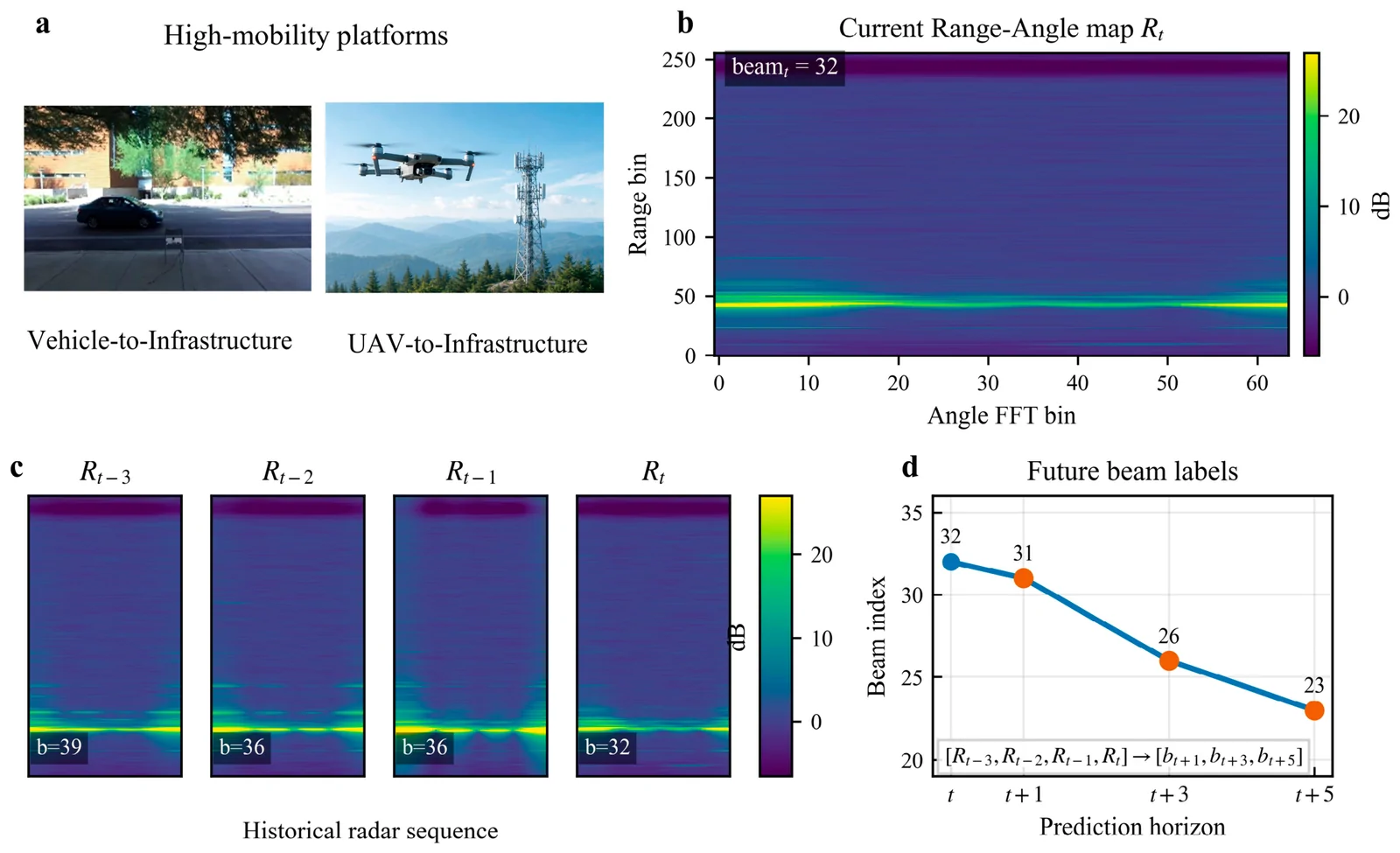
Illustration of high-mobility mmWave beam prediction tasks. (**a**) RGB scene image for visualization only; (**b**) current range-angle map; (**c**) four-frame historical radar sequence; (**d**) current and future beam labels at h = 1, 3, and 5.

**Figure 3:**
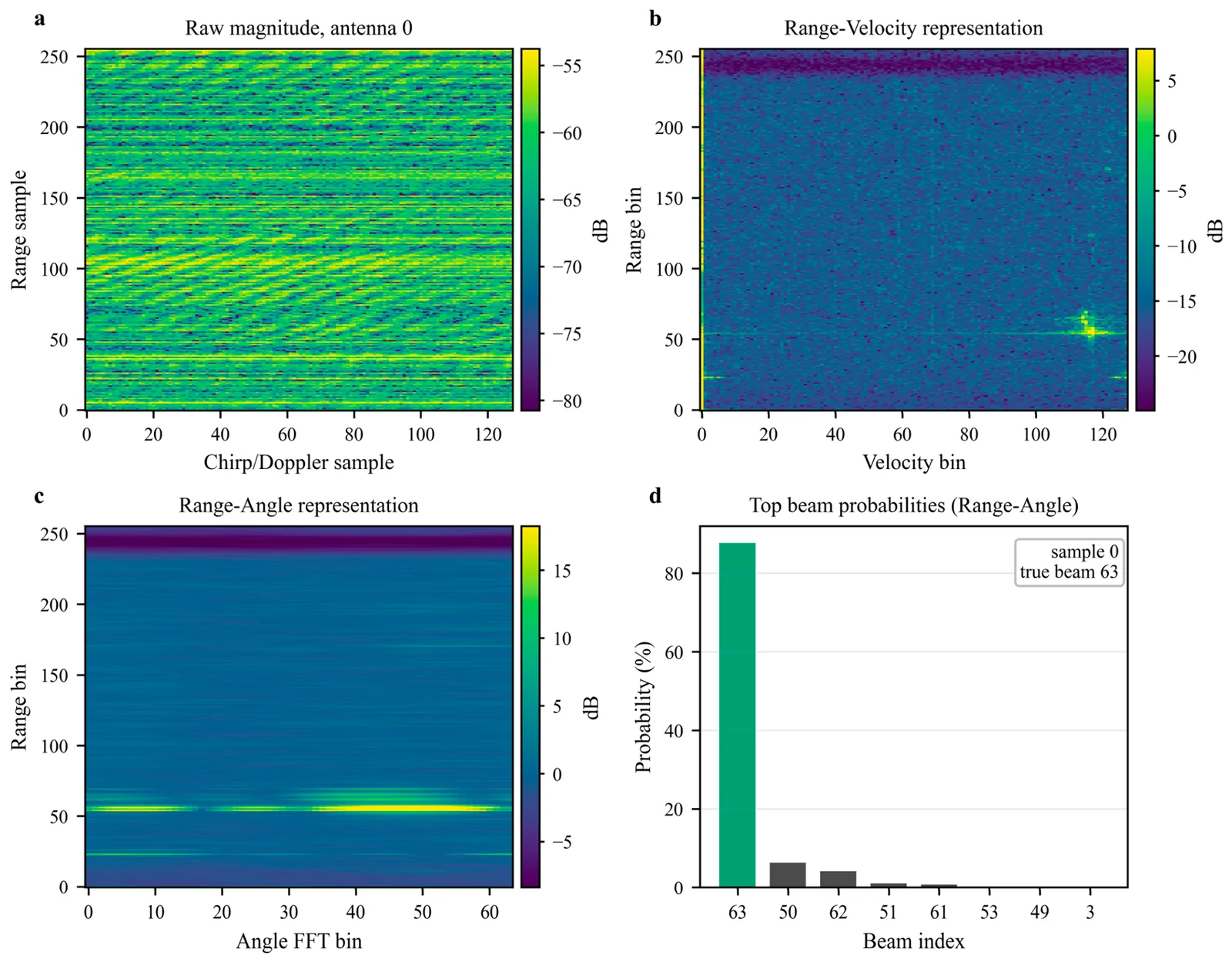
Radar feature processing and beam prediction example. (**a**) Raw radar magnitude slice; (**b**) range-velocity representation; (**c**) range-angle representation; and (**d**) predicted beam probabilities from a range-angle classifier.

**Figure 4:**
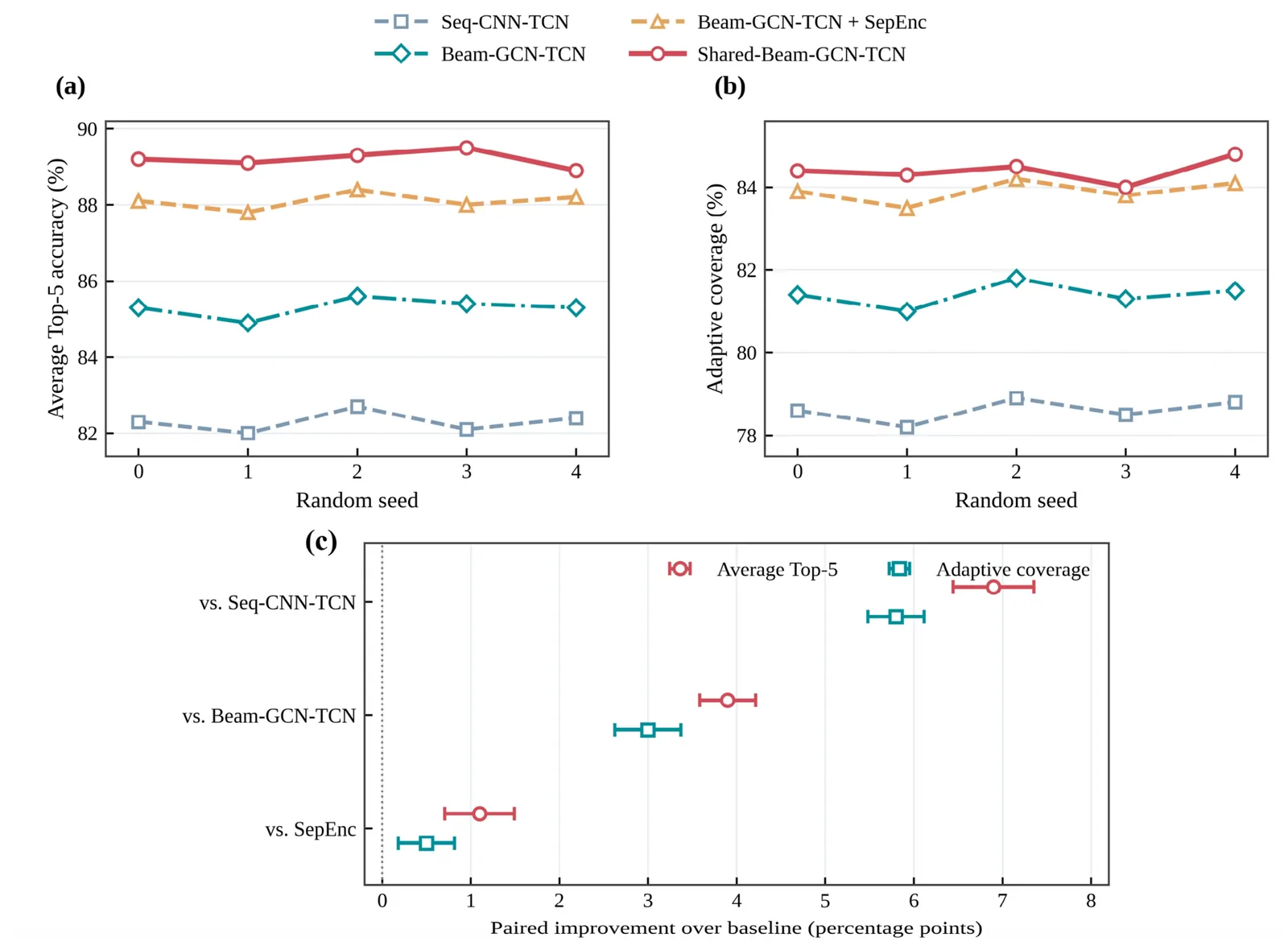
Robustness evaluation of representative beam prediction models across five random seeds. (**a**) Average Top-5 accuracy; (**b**) adaptive beam-search coverage; (**c**) paired performance improvements of Shared-Beam-GCN-TCN over competing models with 95% confidence intervals.

**Figure 5:**
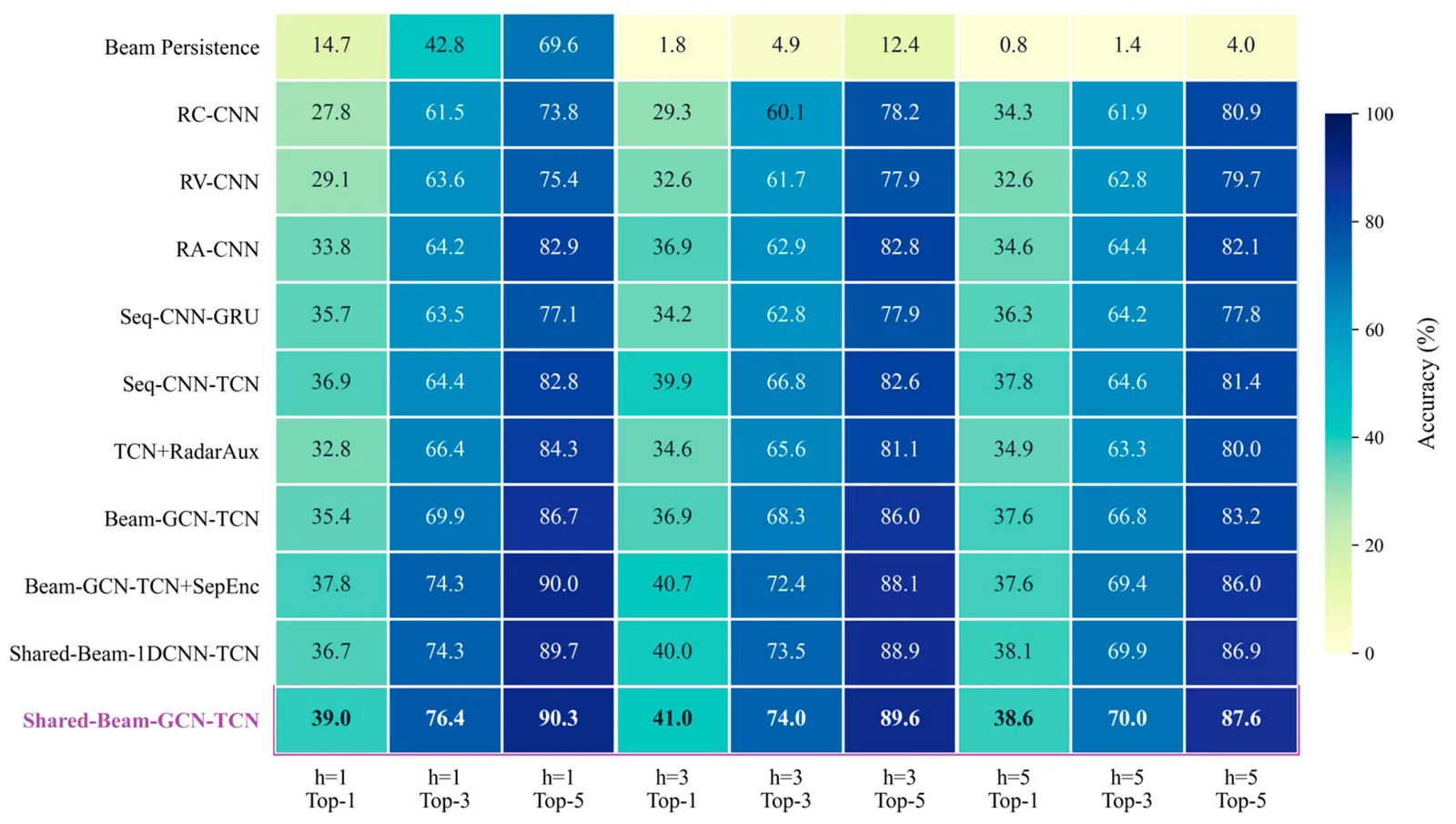
Top-k accuracy as a function of candidate-set size for h = 1, 3, and 5.

**Figure 6:**
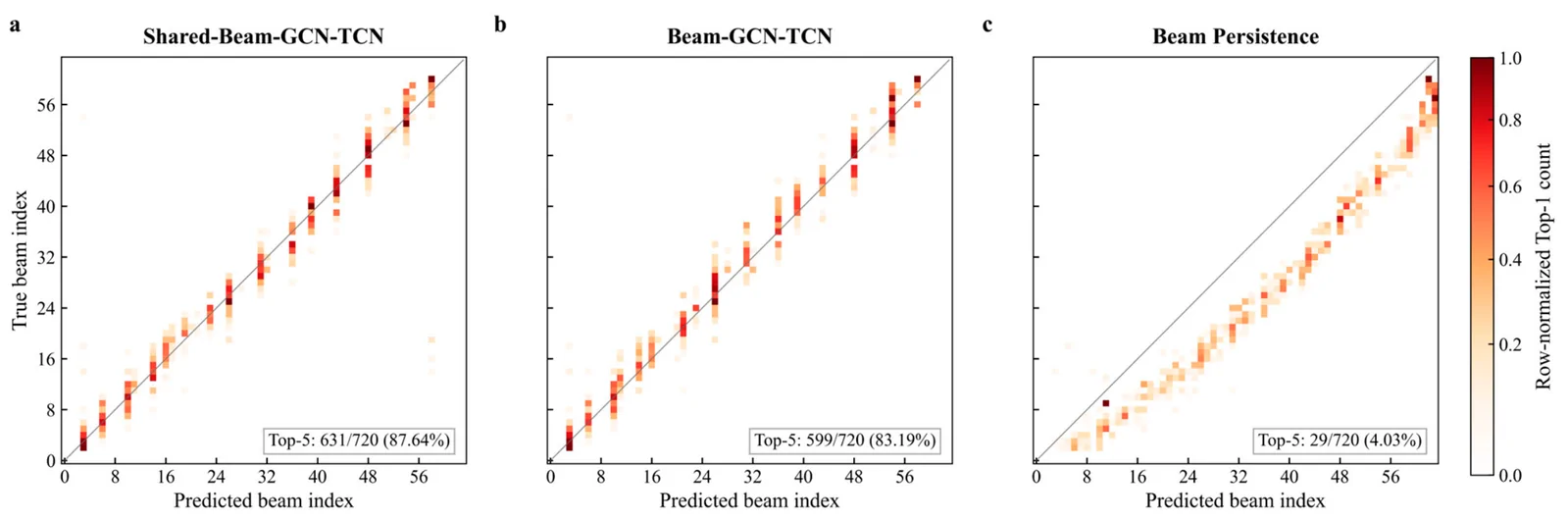
Row-normalized confusion matrices at h = 5 for (**a**) Shared-Beam-GCN-TCN, (**b**) Beam-GCN-TCN, and (**c**) Beam Persistence.

**Figure 7:**
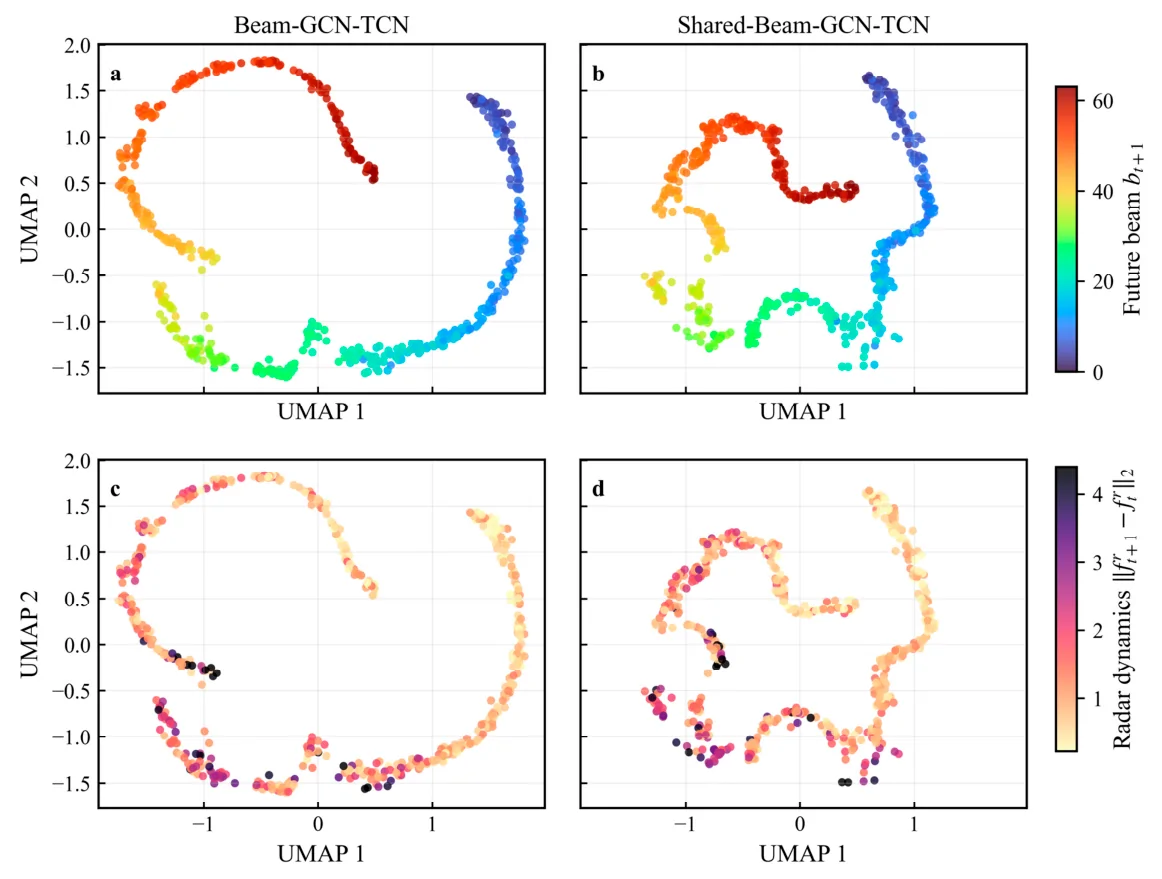
UMAP visualization of temporal latent representations. (**a**) Beam-GCN-TCN colored by future beam index; (**b**) Shared-Beam-GCN-TCN colored by future beam index; (**c**) Beam-GCN-TCN colored by radar dynamics magnitude; (**d**) Shared-Beam-GCN-TCN colored by radar dynamics magnitude.

**Figure 8:**
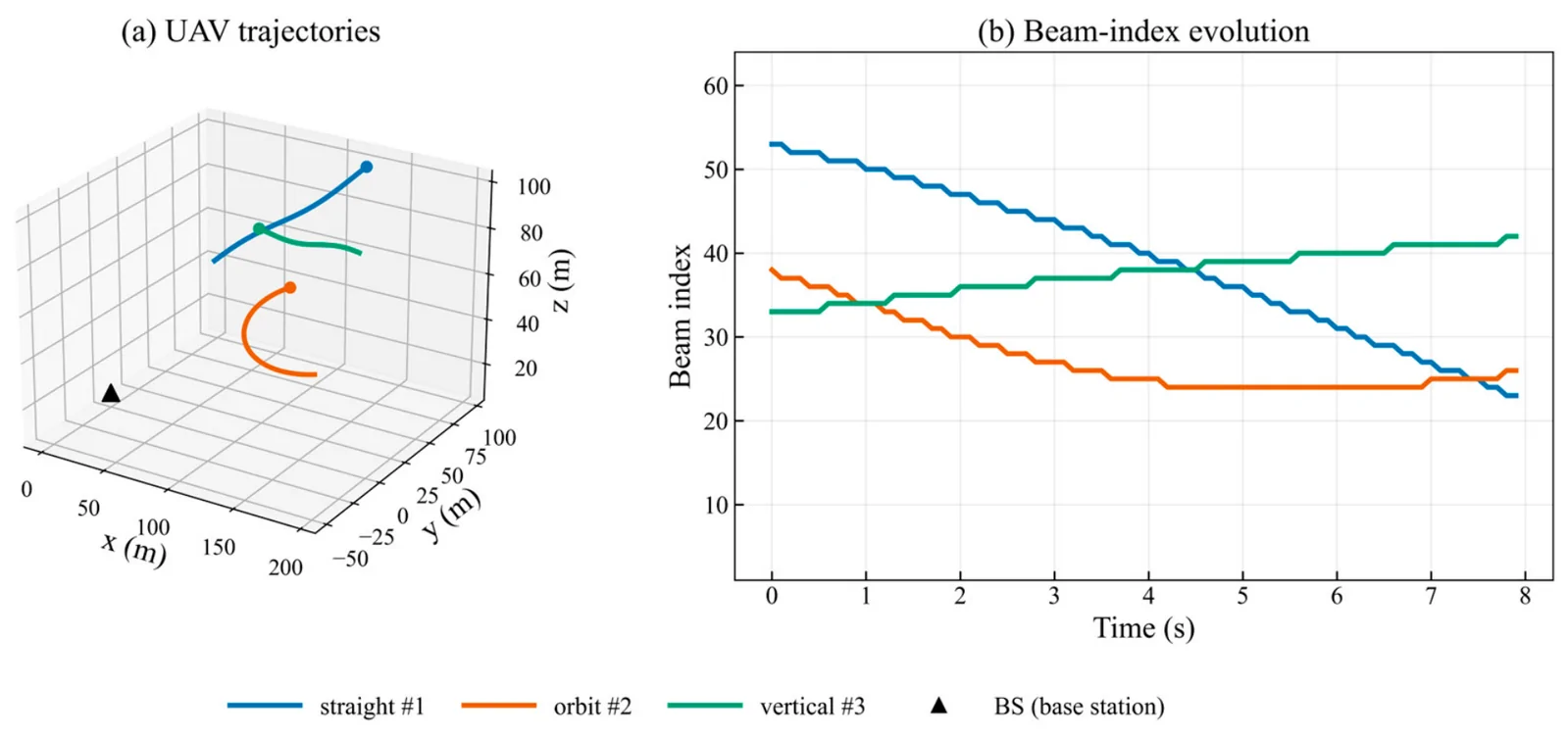
Representative UAV flight trajectories and associated beam-index variations over time. (**a**) Representative UAV flight trajectories; (**b**) future beam-index evolution under different UAV mobility patterns.

**Table 1:** Architecture configuration of the proposed shared radar-dynamics beam prediction network.

Layers/Modules	Parameters	Values
Input	Radar sequence size	4 × 1 × 256 × 64
Input representation	Feature type	2D radar projection (range-angle map)
Frame CNN	Conv channels	1 → 8 → 16 → 32 → 32
Frame CNN	Kernel/pooling	3 × 3/AvgPool 2 × 2
Frame CNN	Adaptive pooling	4 × 4
Frame CNN	Embedding dimension	64
Temporal encoder	Module type	Dilated causal TCN
Temporal encoder	Channels/kernel/dilation	[64, 64]/3/[1, 2]
Shared representation	Dimension	64
Beam-GCN	Beam graph size/average neighbors	64/2
Beam-GCN	Hidden dimension/layers	16/2
Beam heads	Prediction horizons	h = {1, 3, 5}
Future radar dynamics head	Prediction horizon/output dimension	h = 1/64
Regularization	Dropout ratio	0.2

**Table 2:** Dataset and task configurations for different mobility scenarios.

Parameter	Vehicular Scenario	UAV Scenario
Dataset	DeepSense 6G Scenario 9	UAV simulation dataset
Complete trajectories/runs (train/val/test)	22/5/6	50/11/11
Communication scenario	Vehicle-to-Infrastructure (V2I)	UAV-to-Infrastructure (U2I)
Mobility type	Ground vehicle trajectory	3D flight trajectory
Input representation	Range-angle map	Range-angle map
Frame size	1 × 256 × 64	1 × 256 × 64
History length	4 frames	4 frames
Prediction horizons	h = 1, 3, 5	h = 1, 3, 5
Beam classes	64	64
Split protocol	Trajectory-disjoint	Flight-trajectory-disjoint
Synchronized capture rate/interval	7.67 FPS average/0.130 s	10 Hz/0.100 s
Four-frame history span	0.391 s (three intervals)	0.300 s (three intervals)
Prediction lead times (h = 1/3/5)	0.130/0.391/0.652 s	0.100/0.300/0.500 s
Sliding-window stride	1 frame	1 frame
Temporal samples (train/val/test)	3486/670/720	3600/792/792

**Table 3:** Performance comparison of benchmark beam-prediction methods across different prediction horizons.

Method	h = 1 Top-1	h = 1 Top-3	h = 1 Top-5	h = 3 Top-1	h = 3 Top-3	h = 3 Top-5	h = 5 Top-1	h = 5 Top-3	h = 5 Top-5
Beam Persistence	0.147	0.428	0.696	0.018	0.049	0.124	0.008	0.014	0.040
RC-CNN	0.278	0.615	0.738	0.293	0.601	0.782	0.343	0.619	0.809
RV-CNN	0.291	0.636	0.754	0.326	0.617	0.779	0.326	0.628	0.797
RA-CNN	0.338	0.642	0.829	0.369	0.629	0.828	0.346	0.644	0.821
Seq-CNN-GRU	0.357	0.635	0.771	0.342	0.628	0.779	0.363	0.642	0.778
Seq-CNN-TCN	0.369	0.644	0.828	0.399	0.668	0.826	0.378	0.646	0.814
Beam-GCN-TCN	0.354	0.699	0.867	0.369	0.683	0.860	0.376	0.668	0.832

**Table 4:** Ablation results of the proposed architectural components.

Method	h = 1 Top-1	h = 1 Top-3	h = 1 Top-5	h = 3 Top-1	h = 3 Top-3	h = 3 Top-5	h = 5 Top-1	h = 5 Top-3	h = 5 Top-5
TCN + RadarAux	0.328	0.664	0.843	0.346	0.656	0.811	0.349	0.633	0.800
Beam-GCN-TCN	0.354	0.699	0.867	0.369	0.683	0.860	0.376	0.668	0.832
Beam-GCN-TCN + SepEnc	0.378	0.743	0.900	0.407	0.724	0.881	0.376	0.694	0.860
Shared-Beam-CNN-TCN	0.367	0.743	0.897	0.400	0.735	0.889	0.381	0.699	0.869
Shared-Beam-GCN-TCN	0.390	0.764	0.903	0.410	0.740	0.896	0.386	0.700	0.876

**Table 5:** Statistical comparison of representative baseline models across five random seeds.

Method	Avg. Top-5 (%)	Adaptive Coverage (%)
Seq-CNN-TCN	82.30 ± 0.27	78.60 ± 0.27
Beam-GCN-TCN	85.30 ± 0.25	81.40 ± 0.29
Beam-GCN-TCN + SepEnc	88.10 ± 0.22	83.90 ± 0.27
Shared-Beam-GCN-TCN	89.20 ± 0.22	84.40 ± 0.29
Difference vs. Seq-CNN-TCN (pp)	+6.90 [6.44, 7.36]	+5.80 [5.48, 6.12]
Difference vs. Beam-GCN-TCN (pp)	+3.90 [3.58, 4.22]	+3.00 [2.63, 3.37]
Difference vs. SepEnc (pp)	+1.10 [0.71, 1.49]	+0.50 [0.18, 0.82]

**Table 6:** Average performance and adaptive beam-search efficiency across prediction horizons.

Method	Top-1	Top-3	Top-5	Beam Error	Adaptive Accuracy	Avg. Searched Beams
Beam Persistence	0.058	0.163	0.287	5.112	0.057	1.000
RC-CNN	0.305	0.612	0.776	1.636	0.780	4.088
RV-CNN	0.314	0.627	0.777	1.657	0.743	4.120
RA-CNN	0.351	0.638	0.826	1.440	0.789	3.871
Seq-CNN-GRU	0.354	0.635	0.776	1.654	0.783	4.725
Seq-CNN-TCN	0.382	0.653	0.823	1.433	0.786	4.331
Beam-GCN-TCN	0.367	0.683	0.853	1.480	0.814	4.190
TCN + RadarAux	0.341	0.651	0.818	1.562	0.763	4.265
Beam-GCN-TCN + SepEnc	0.387	0.720	0.881	1.317	0.839	4.167
Shared-Beam-CNN-TCN	0.382	0.726	0.885	1.339	0.822	3.882
Shared-Beam-GCN-TCN	0.395	0.735	0.892	1.271	0.844	4.019

**Table 7:** Future radar-feature prediction and retrieval performance.

Method	Cosine Similarity ↑	Retrieved Beam Error ↓	Within-One-Beam ↑
Shared radar prediction	0.959	1.73	55.08%
Current-feature persistence	0.966	1.90	42.38%
Linear feature extrapolation	0.917	2.17	47.07%

**Table 8:** Comparison of future-beam prediction performance under the UAV mobility scenario.

Method	Top-1	Top-3	Top-5	Beam Error
Seq-CNN-TCN	0.2652	0.7146	0.8822	1.347
Beam-GCN-TCN	0.2782	0.7180	0.9242	1.166
Shared-Beam-GCN-TCN	0.3123	0.7908	0.9339	1.022

## Data Availability

The dataset used in this study is publicly available from the DeepSense 6G dataset repository at https://www.deepsense6g.net/ (accessed on 11 October 2026). The experiments were conducted using Scenario 9 of the DeepSense 6G dataset, which provides synchronized radar measurements and mmWave communication beam labels. The processed datasets and implementation details generated during the current study are available from the corresponding author upon reasonable request.
